# Integration analysis of microRNAs as potential biomarkers in early-stage lung adenocarcinoma: the diagnostic and therapeutic significance of miR-183-3p

**DOI:** 10.3389/fonc.2024.1508715

**Published:** 2024-12-17

**Authors:** Guodong Huang, Yuxia Liu, Lisha Li, Bing Li, Ting Jiang, Yufeng Cao, Xiaoping Yang, Xinning Liu, Honglin Qu, Shitao Li, Xin Zheng

**Affiliations:** ^1^ Central Laboratory, Qingdao Hiser Hospital Affiliated of Qingdao University (Qingdao Hospital of Traditional Chinese Medicine), Qingdao, Shandong, China; ^2^ Department of Respiration, Second Affiliated Hospital of Shandong University of Traditional Chinese Medicine, Jinan, Shandong, China; ^3^ Department of Respiration, Qingdao Hiser Hospital Affiliated of Qingdao University (Qingdao Hospital of Traditional Chinese Medicine), Qingdao, Shandong, China; ^4^ Cancer Center, Qingdao Hiser Hospital Affiliated of Qingdao University (Qingdao Hospital of Traditional Chinese Medicine), Qingdao, Shandong, China

**Keywords:** lung adenocarcinoma, microRNA sequencing, biomarkers, miR-183-3p, SESN1

## Abstract

**Introduction:**

Lung adenocarcinoma (LUAD) poses a significant therapeutic challenge, primarily due to delayed diagnosis and the limited efficacy of existing treatments.

**Methods:**

To understand the pathogenesis and identify diagnostic biomarkers for LUAD in the early stage, we investigated differential miRNA expression in 33 stage I LUAD patients between tumor and matched paracancerous tissues by Illumina Sequencing. Target genes of differentially expressed miRNAs were predicted using TargetScan and miRDB databases and further analyzed by GO and KEGG pathway enrichment analysis. The miRNAs expression results were verified using qRT-PCR. Additionally, we evaluated the clinical significance of miRNAs by the TCGA database. miR-183-3p was chosen for subsequent biological functional studies by cell proliferation assays, cell migration and cell invasion assays, cell apoptosis and cell cycle assays in LUAD cells. The clinical relevance target genes of miR-183-3p were predicted by TargetScan databases and bioinformatics assays. Gene-specific experimental validation was performed using qRT-PCR, western blotting and luciferase reporter assays.

**Results:**

We identified 36 differentially expressed miRNAs between LUAD tissues and matched paracancerous tissues. Target genes for these miRNAs revealed associations with processes and pathways such as RNA biosynthesis, intracellular signaling, protein transport, and the Ras, MAPK, and PI3K-AKT pathways. The qRT-PCR results were in alignment with the sequencing data for 19 out of these 21 miRNAs which not yet implicated in LUAD, 13 were up-regulated, 6 were down-regulated. The clinical relevance assays showed that 5 up-regulated miRNAs have diagnostic value for LUAD. miR-183-3p showed significant advantages in the result of sequencing, qRT-PCR, and clinical relevance assay. Biological functional assays showed that miR-183-3p emerged as a key regulator, promoting LUAD cell proliferation, decreasing apoptosis, and augmenting migration and invasion capabilities. The clinical relevance assays and experimental validation showed SESN1 as a clinical significance target of miR-183-3p.

**Discussion:**

Our study lays the foundation for investigating miRNAs with diagnostic significance in early-stage LUAD, pointing out that inhibition of miR-183-3p may serve as a novel therapeutic in LUAD.

## 1 Introduction

Lung cancer continues to be a major public health challenge, ranking as the leading cause of cancer-related mortality worldwide and the second most common cancer, according to the GLOBOCAN database (1). Non-small-cell lung cancer (NSCLC), accounting for approximately 85% of all cases, is primarily divided into two histological subtypes: lung adenocarcinoma (LUAD) and lung squamous carcinoma (LUSC) (2). Although there have been strides in LUAD treatments, the outlook for patients with advanced stages of the disease remains bleak. The sobering statistics show an average 5-year survival rate of a mere 15%, with a mortality rate reaching 85% (3). A significant proportion of patients receive their diagnosis at an advanced stage, with half not surviving past the first year post-diagnosis. This underscores the urgent imperative for exploring effective diagnosis and treatment biomarkers in early-stage LUAD.

Non-coding RNAs, especially microRNAs (miRNAs) and circular RNAs (cirRNAs), profoundly influence the tumor microenvironment. These RNA molecules play vital roles in various cellular processes, including cell differentiation, proliferation, immune regulation, and apoptosis in tumors (4). In this context, miRNAs, small non-coding RNA sequences of 18-25 nucleotides, are notable for their capacity to inhibit translation initiation or induce mRNA degradation by specifically binding to the 3’ untranslated region of target mRNAs. A burgeoning body of research suggests that dysregulated miRNAs play pivotal roles in numerous human conditions, functioning as either on-cogenes or tumor suppressors across various cancers, and can be used as a marker of tumor diagnosis and prognosis (5). To identify miRNAs with potential as biomarkers for early LUAD diagnosis, we employed Illumina next-generation sequencing to examine miRNA expression differences in LUAD tumors and adjacent normal tissues in 33 early-stage LUAD patients, complemented by comprehensive bioinformatics and *in vitro* experiments.

The miR-183 cluster, which includes miR-96, miR-182, and miR-183, is increasingly recognized as a significant regulatory nexus in several cancers, notably LUAD (6). Nevertheless, the specific roles of the miR-183 family in different cancers, including lung cancer, are still under debate. For instance, miR-183-5p is posited to act as a tumor suppressor in lung cancer through its inhibitory action on PIK3CA (7). Concurrently, another research emphasizes miR-183-5p’s pro-tumorigenic role in NSCLC by inhibiting LOXL4 (8) and PTEN (9). Similar disputes also appear in the study of miR-183-3p. miR-183-3p inhibits cell proliferation and migration, and induces apoptosis in prostate cancer cells (10). In another study, overexpression of miR-183-3p was associated with poor survival in female LUAD non-smokers, so miR-183-3p is put forth as a prognostic marker for LUAD in female non-smokers (11). Overall, whether mir-183-3p plays a pro-cancer or anti-cancer role in cancers is controversial, and the target genes and the mechanism in LUAD have not been ascertained. According to the above situation, our research cleared the miR-183-3p comprehensive effect and specific target gene in LUAD.

In our pursuit to understand miRNA expression profiles and their roles in lung cancer pathogenesis, we embarked on an exhaustive study. We aimed to discern differentially expressed miRNAs, craft specific miRNA-mRNA regulatory networks for LUAD, and undertake functional enrichment analyses of the implicated target genes. Based on Illumina next-generation sequencing and qRT-PCR, miR-183-3p manifested the most notable differential expression and showed significant diagnostic implications. Our findings demonstrate that miR-183-3p promotes proliferation, migration, and invasion in the A549 and H1975 cell lines, and inhibition of miR-183-3p is the potential treatment of LUAD. Moreover, SESN1 is the target gene with clinical significance of miR-183-3p. Through our research, we furnish pivotal insights and lay a solid groundwork for the deeper exploration of pathogenesis and the identification of potential diagnostic markers and therapeutic targets for early-stage LUAD.

## 2 Materials and methods

### 2.1 Tissue specimen collection

Lung cancer tissues and adjacent tissues (7 cm away from the cancer tissue) were collected from 33 stage I LUAD patients in Qingdao Hiser Hospital Affiliated of Qingdao University (Qingdao Traditional Chinese Medicine Hospital) in 2021. These patients, aged between 18 and 75 years, were diagnosed with LUAD by pathological diagnosis for the first time. After obtaining consent from the patients’ families and approval from the ethics committee, the tissues were promptly isolated and preserved in RNA preservation solution, then stored at −80**°**C. The studies involving human participants were reviewed and approved by the Ethics Committee of Qingdao Hiser Hospital Affiliated of Qingdao University (Qingdao Traditional Chinese Medicine Hospital) (2020HC03LS001, March 25, 2020). The patients provided their written informed consent to participate in this study.

### 2.2 Total RNA extraction, quality control, cDNA library construction, and Illumina sequencing

RNA extraction samples were stored at -80°C. Total RNA was extracted from 30 mg of tissue using a trizol reagent (Invitrogen, California, United States). The RNA samples’ content and quality were evaluated using the RNA Nano 6000 kit (Agilent Technologies, California, United States), and RNA quantification was conducted with an ND-2000 spectrophotometer. The RNA samples used for sequencing library construction were required to meet the following criteria: OD260/280 = 1.8-2.2, OD260/230 ≥ 2.0, RIN ≥ 6.5, 28S: 18S ≥ 1.0. RNA integration and RIN number detection were performed using an Agilent bioanalyzer (Agilent Technologies, California, United States). The Illumina TruSeq Small RNA kit (Illumina, California, United States) was used to construct 12 libraries, which were subsequently sequenced on an Illumina HiSeq 2500 sequencing platform (Illumina, California, United States).

### 2.3 Differential expression analysis of miRNAs

To identify differentially expressed miRNAs in LUAD and paracancerous tissues, the Blast software was employed to annotate the miRNAs using the Rfam database. Reference genome alignment of miRNA sequences without non-miRNA sequences (e.g., rRNA, snoRNA, snRNA, and tRNA) was compared to miRNAs in the miRBase database (miRBase2.0, http://www.mirbase.org/). The identified miRNAs were considered known miRNAs. The signature hairpin structure of miRNA precursors was used to predict novel miRNAs. The miRNAs were aligned to the reference genome, and surrounding sequences were extracted for secondary structure prediction. Novel miRNAs were identified based on the prediction results, combined with dicer restriction site information and energy value. The expression profile of all miRNAs was obtained, and differential expression analysis of known miRNAs and novel predicted miRNAs between samples was performed by setting fold change (FC) ≥ 1 and *p* ≤ 0.05. Differentially expressed miRNAs were screened as follows: (1) Up-regulated when *p* ≤ 0.05 and Log_2_(FC) ≥ 1; (2) Down-regulated when p ≤ 0.05 and Log_2_(FC) ≤ −1.

### 2.4 miRNA target genes prediction and functional enrichment

Target genes of significantly differentially expressed miRNAs were predicted based on the TargetScan and miRDB databases. The Gene Ontology (GO) enrichment database and Kyoto Encyclopedia of Genes and Genomes (KEGG) database were employed for online bioinformatics analysis of the differential genes. Up-regulated and down-regulated differentially expressed genes were mapped to the GO database (http://www.geneontology.org/) and enriched at the biological process, cellular component, and molecular function levels, respectively. KEGG pathway enrichment analysis was conducted using KOBAS (http://kobas.cbi.pku.edu.cn/) to annotate and enrich the signaling pathways of the differential genes.

### 2.5 Protein-protein interaction network

The target genes of differentially expressed miRNAs were predicted using the TargetScan (www.targetscan.org/) and miRDB (www.mirdb.org/miRDB/) analysis tools. The miRNA-mRNA networks were visualized using the Cytoscape platform software. PPI information was assessed using STRING version 10.5 (string-db.org), and a confidence score > 0.9 was set as the cut-off criterion. Significant modules were selected from the PPI network using the CytoNCA application in Cytoscape.

### 2.6 Verification of miRNAs expression and target gene expression by real-time gluorescence quantitative polymerase chain reaction

The reliability of transcriptome sequencing results was verified by qRT-PCR. Total RNA from A549 and Beas-2B cells was isolated using an RNAprep Pure Cell kit (TianGen, Beijing, China), followed by cDNA synthesis using PreScript III RT ProMix (EnzyValley, Guangzhou, China). Subsequently, qPCR was performed with 2× Universal qPCR SYBR Green Master Mix (Low Rox) (Meilunbio). The specific primer sequences are listed in **Supplementary Table 1**. Three replicates were set for each sample, and the reaction program was as follows: an initial denaturation at 95**°**C for 1 min, followed by 45 amplification cycles, each consisting of 10 s at 95**°**C and 30 s at 60**°**C. The relative quantitative results were calculated using the 2^-ΔΔCt^ method.

### 2.7 Expression of miRNA and their related clinical significance in LUAD

We first obtained paired and unpaired expression differences of miRNAs in tumor and adjacent normal tissues from The Cancer Genome Atlas (TCGA) database (https://portal.gdc.cancer.gov). Data were evaluated in the R software v3.6.3, and visualized using the ggplot2 (v3.3.2) software package. To investigate the clinical significance of miRNA expression in LUAD, we utilized the Kaplan−Meier Plotter database (http://kmplot.com/analysis/) to analyze the relationship between miRNA expression and clinical endpoints, including disease-specific survival (DSS) and progression-free interval (PFI). Additionally, to evaluate the diagnostic performance of miRNA in LUAD, we employed receiver operating characteristic (ROC) curves. The area under the curve (AUC) was used to assess the accuracy level, with AUC values classified as low (0.5-0.7), moderate (0.7-0.9), and high (above 0.9), with an AUC closer to 1 indicating superior diagnostic efficacy.

### 2.8 Cell lines and cell culture

Beas-2B, A549, and H1975 cell lines were purchased from Meilunbio (Dalian, China) and subsequently authenticated through short tandem repeat (STR) analysis. Beas-2B cells were cultured in DMEM medium (Gibco, Massachusetts, United States), A549 cells were cultured in F-12K medium (Gibco), and H1975 cells were cultured in RPMI-1640 medium (Gibco). All cell lines were supplemented with 10% fetal bovine serum (Gibco) and incubated in a fully humidified incubator (ESCO Technologies Inc., Missouri, United States) at 37°C with 5% CO^2^.

### 2.9 Cell transient transfection

To modulate miR-183-3p expression, we acquired a negative control (NC) mimic, miR-183-3p mimic, NC inhibitor, and miR-183-3p inhibitor from GenePharma (Genepharma Biotechnology, Shanghai, China). We performed transient transfections on A549 and H1975 cells using GP-transfect-Mate (Genepharma Biotechnology) according to the manufacturer’s specifications. The efficiency of transient transfection was evaluated using qRT-PCR.

### 2.10 Cell proliferation assays

Cell proliferation was assessed using Cell Counting Kit-8 (CCK-8) assays (Meilunbio). A549 and H1975 cells were cultured in 96-well plates and transfected with either a mimic or inhibitor for 24, 48, or 72 hours. Following the addition of 10 μl of CCK-8 reagent per well, the plates were incubated at 37**°**C for 2 hours. Subsequently, the optical density at 450 nm was measured using a Victor Nivo Multimode Microplate Reader (PerkinElmer, Massachusetts, United States). All experiments were conducted in triplicate.

### 2.11 Cell migration and cell invasion assays

Wound-healing assays were conducted to assess cell migration. Pipettes were used to create scratches, and cells were transfected with mimic or inhibitor for and 48 h. Photographs were captured using a Nikon Eclipse Ti2 inverted microscope (Nikon, Tokyo, Japan), and the wound area was measured using ImageJ software (National Institutes of Health, Maryland, United States).

Cell invasion assays were evaluated using 8 μm pore size transwell insert chambers (Biofil, Guangzhou, China) coated with matrigel (Corning, New York, United States). A549 and H975 cells (1.5 × 10^5^/well) were seeded in the upper chamber with serum-free medium, while 20% FBS medium was added to the lower chamber. After 48 h, non-invading cells in the upper chamber were removed by cotton swabs, and invading cells were fixed with 4% paraformaldehyde (Biosharp, Hefei, China) for 20 min and stained with 0.1% crystal violet (Solarbio, Beijing, China) for 15 min. The stained cells were photographed using a Nikon Eclipse Ti2 inverted microscope (Nikon, Tokyo, Japan).

### 2.12 Cell apoptosis and cell cycle assays

A549 and H1975 cells were seeded in 6-well plates, incubated overnight, and then transiently transfected with mimic or inhibitor for 48 h. Cells were washed with pre-cooled PBS and then centrifuged, collecting a sample of 1 × 10^6^ cells. Apoptosis was assessed using an Annexin V-FITC/PI Apoptosis Kit (Multi Sciences, Hangzhou, China). Each cell group was stained with 5 μl of Annexin V-FITC and 10 μl of propidium iodide (PI) solution, incubated in the dark at room temperature for 5 mins and then analyzed using flow cytometry (Beckman Coulter Biotechnology, California, United States). The apoptosis rate was determined by quantifying the proportion of cells in different stages of apoptosis. This involved identifying early apoptotic cells (Annexin V-FITC positive and PI negative) and late apoptotic or necrotic cells (positive for both Annexin V-FITC and PI). The apoptosis rate was calculated as follows: Cell Apoptosis Rate (%) = [(Number of Early Apoptotic Cells + Number of Late Apoptotic/Necrotic Cells)**/**Total Number of Cells] × 100. Cell cycle experiments were performed using a Cell Cycle Staining Kit (Multi Sciences). The cells of each group were added 1 ml of DNA Staining solution and 10 μl of Permeabilization solution, incubated in the dark at room temperature for 30 min, and then measured by Cytoflex Flow Cytometry (Beckman Coulter Biotechnology). The distribution of cells in the G1, S, and G2/M phases was determined using FlowJo software (BD Bioscience, California, United States).

### 2.13 Western blot analysis

For Western blot analysis, 30 μg of whole-cell protein lysates were separated using 10% denatured polyacrylamide slab gels (Boster Biological Technology, California, United States) and subsequently electroblotted onto polyvinylidene fluoride membranes (Merck Millipore, Massachusetts, United States). The membranes were incubated with antibodies against β-Tubulin, Caspase-3, Bax, Bcl-2, SESN1, and GAPDH (Boster Biological Technology), followed by incubation with secondary antibodies (Meilunbio). Detection was carried out using an ECL luminescence reagent (Meilunbio), and the antibody luminescence was captured using a ChemiDoc XRS+ imaging system (Bio-Rad, California, United States). Relative quantitative analysis of the protein bands was performed using ImageJ software (National Institutes of Health).

### 2.14 Dual-luciferase reporter assays

Dual-luciferase reporter assays were conducted to investigate the potential targeting relationship between SESN1 and miR-183-3p. Initially, a pGL3-SESN1 WT reporter plasmid, which contained the 3’ UTR of SESN1 with potential binding sites for miR-183-3p, was constructed. Additionally, a mutant form, pGL3-SESN1 MUT, was generated by mutating the potential miR-183-3p binding sites. These luciferase plasmids were co-transfected into LUAD cells along with overexpressed miR-183-3p plasmid and pRL-TK (an internal reference plasmid expressing Renilla luciferase). After 48 h of transfection, luciferase activity was measured using a dual-luciferase reporter assay system (Meilunbio). The relative luciferase activity was determined based on the ratio of firefly luciferase to Renilla luciferase. This experiment was conducted in triplicate.

### 2.15 Statistical analysis

Graph production, data distribution, and statistical analyses were performed using GraphPad Prism 8. Analysis of variance (ANOVA) or t-tests was used to investigate significant differences between indicated groups. *p < 0.05, **p < 0.01, ***p < 0.01 were considered statistically significant, and ns represents statistically non-significant.

## 3 Results

### 3.1 Differential expression of miRNAs in early-stage LUAD tissues

**Figure 1:**
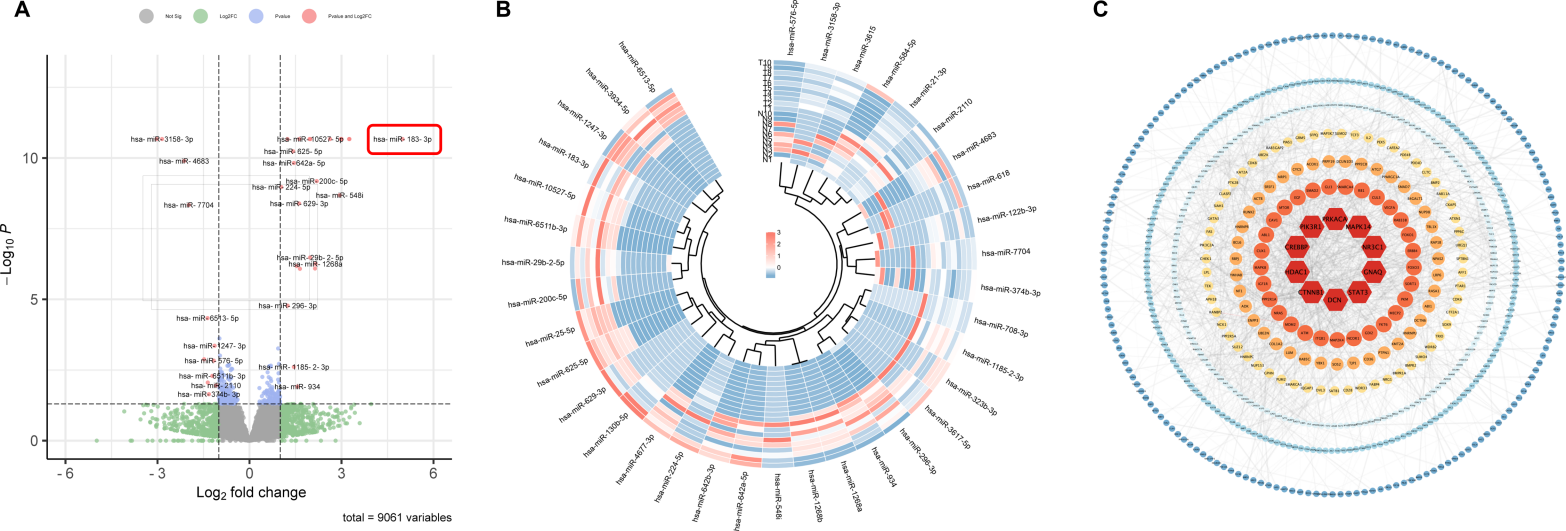
Differential expression of miRNAs in early-stage LUAD tissues. **(A)** Volcano plots of known differentially expressed miRNAs in lung cancer and adjacent tissues using fold change ≥ 1 and *p* ≤ 0.05. **(B)** Heat map of known differentially expressed miRNAs in lung cancer and adjacent tissues. **(C)** PPI network of differentially expressed genes in lung cancer. The top 10 hub genes are shown in deep red.

To comprehensively understand the pathogenesis and pinpoint diagnostic biomarkers of LUAD, we embarked on an extensive miRNA profiling study. Utilizing Illumina next-generation sequencing, we probed the variations of 33 patients with stage-I LUAD in miRNA expression between LUAD tumors and their adjacent normal tissues. By adopting a stringent cut-off criterion (*p* < 0.05 and |Log_2_FC| > 1.0), we discerned 2831 novel significantly altered miRNAs in tumor tissues compared to the adjacent non-tumor counterparts. Specifically, 1827 of these miRNAs were markedly up-regulated, while 1004 saw significant down-regulation. Furthermore, our research pinpointed 36 known differentially expressed miRNAs: 24 up-regulated and 12 down-regulated. A visual representation of these differentially expressed miRNAs, captured through a volcano plot, is showcased in **Figure 1A**, and the detailed data is shown in **Supplementary Tables 2**, **3**.

It’s worth noting that many miRNAs identified in our analysis have prior associations with LUAD progression. For example, miR-584-5p and miR-934 are known to restrain the proliferation, migration, and invasion attributes of NSCLC (12, 13) Moreover, after knocking down miR-21-3p, lung cancer cells showed heightened sensitivity towards docetaxel and cisplatin treatments (14). The down-regulation of miR-3934-5p has been linked with enhanced cisplatin sensitivity in A549 cells, acting via the TP53INP1 pathway (15). In contrast, an elevated expression of miR-934 is not only an independent prognostic factor but also augments the proliferative, migratory, and invasive tendencies of NSCLC cells (16). The interplay between miR-4677-3p and TTA-AS1 further facilitates tumor growth and metastasis (17)], while the miR-548i/AGO1 axis accelerates NSCLC migration and invasion (18). Our results echo these established findings. For a more detailed visualization, the heatmap illustrating the expression nuances of these miRNAs is provided in **Figure 1B**.

### 3.2 PPI network analysis of target genes in differentially expressed miRNAs

To unravel the intricacies of the functions and interplays among the differentially expressed miRNAs, we employed the TargetScan and miRDB for target gene prediction of the identified differentially expressed miRNAs. This effort yielded 8789 potential target genes. Using the STRING online database, we designed a protein-protein interaction (PPI) network, which was visualized using Cytoscape, encompassing 674 nodes and 1690 connecting edges. Further analysis with CytoNCA allowed us to pinpoint 10 hub genes based on node count in the interaction network and associated p-values. These genes, namely CTNNB1, HDAC1, CREBBP, PIK3R1, PRKACA, MAPK14, NR3C1, GNAQ, STAT3, and DCN, have previous implications in lung cancer, serving as potential early diagnostic and therapeutic biomarkers (19–23). This PPI network is graphically represented in **Figure 1C**, with accompanying details in **Table 1**.

**Table 1:** 10 hub genes screened from PPI network by CytoNCA.

Ranking	Gene	Betweenness
1	CTNNB1	80851.81
2	HDAC1	76563.17
3	CREBBP	44769.29
4	PIK3R1	33577.953
5	PRKACA	27635.137
6	MAPK14	25378.027
7	NR3C1	25318.941
8	GNAQ	23425.621
9	STAT3	23298.322
10	DCN	23252.113

### 3.3 Functional and pathway enrichment analysis of target genes in differentially expressed miRNAs

**Figure 2:**
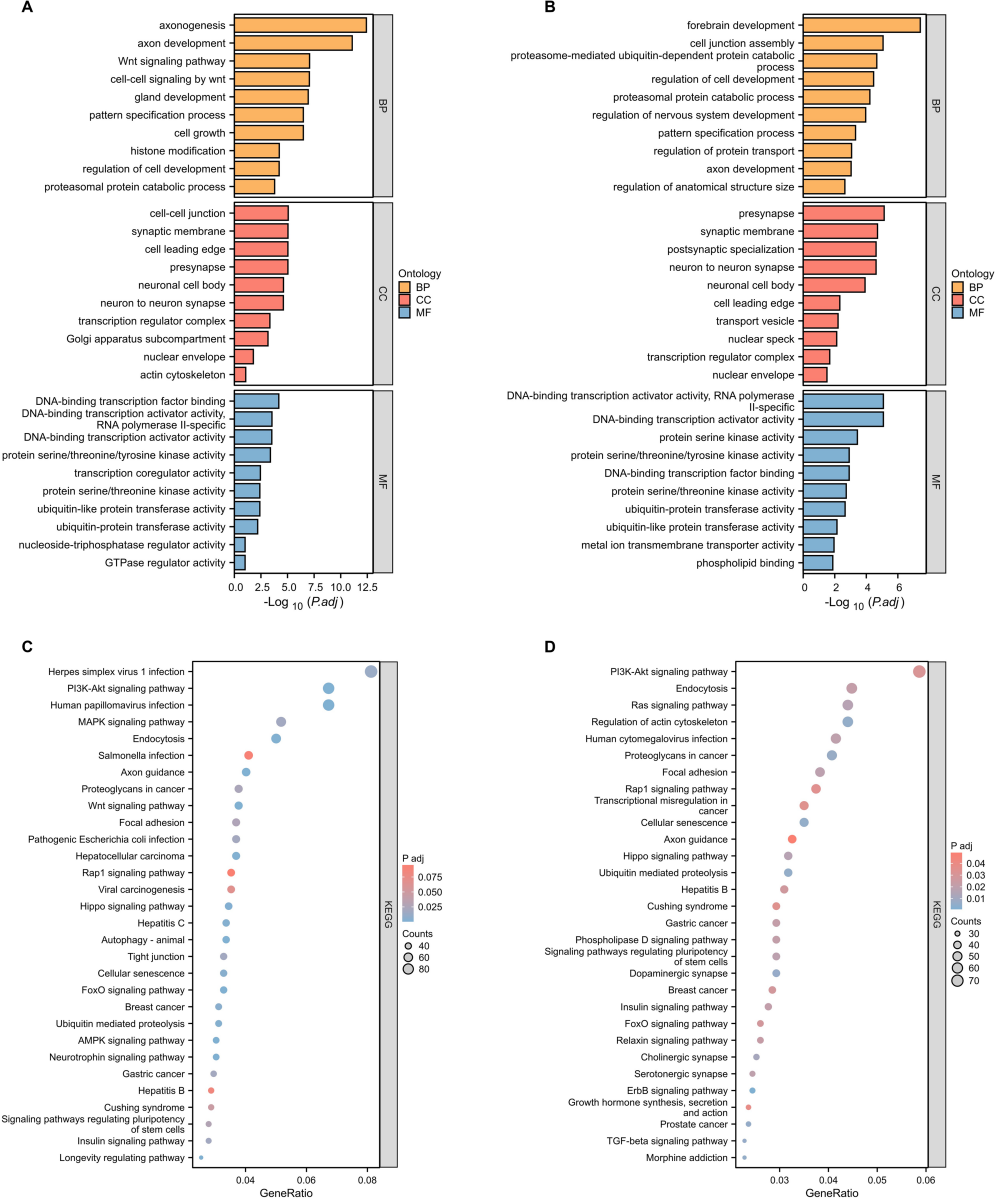
Functional and pathway enrichment analysis of target genes of significantly differentially expressed miRNAs. **(A)** GO functional enrichment analysis of known differentially expressed up-regulated miRNA target genes in lung cancer tissues and adjacent tissues. BP, biological process; CC, cellular component; MF, molecular function. **(B)** GO functional enrichment analysis of known differentially expressed down-regulated miRNA target genes in lung cancer tissues and adjacent tissues. **(C)** KEGG pathway enrichment analysis of known differentially expressed up-regulated miRNA target genes in lung cancer tissues and adjacent tissues. **(D)** KEGG pathway enrichment analysis of known differentially expressed down-regulated miRNA target genes in lung cancer tissues and adjacent tissues.

To elucidate the potential biological roles of the differentially expressed miRNAs in lung cancer tissues, we employed the DAVID tool for GO enrichment analysis of the target genes of these 36 distinct miRNAs. The analysis showcased that the target genes of the 24 up-regulated miRNAs participated in 632 biological processes, 150 cellular components, and 134 molecular functions (**Figure 2A**). Conversely, the target s of the 12 down-regulated miRNAs engaged in 518 biological processes, 151 cellular components, and 128 molecular functions (**Figure 2B**).

Universally, the targets of both the up-regulated and down-regulated miRNAs had affiliations with processes like RNA biosynthesis regulation, kinase activity, and ubiquitin-like protein transferase activity. Distinctively, the genes targeted by up-regulated miRNAs predominantly influenced the Wnt signaling pathway, cell growth, and histone modification. In contrast, those of the down-regulated miRNAs had a more significant association with forebrain development, regulation of nervouse system development, and ubiquitin-dependent protein catabolic.

Of particular note, the PI3K-AKT signaling pathway, well-documented for its pivotal role in lung cancer, emerged prominently. Both the up-regulated and down-regulated miRNA target genes exhibited strong enrichment in this pathway, featuring 82 and 72 target genes, respectively (**Figures 2C, D**). This prominence of the PI3K-AKT signaling pathway corroborates its crucial role in lung cancer progression, aligning with existing literature (24–26).

### 3.4 Validation of differentially expressed miRNA by qRT-PCR

**Figure 3:**
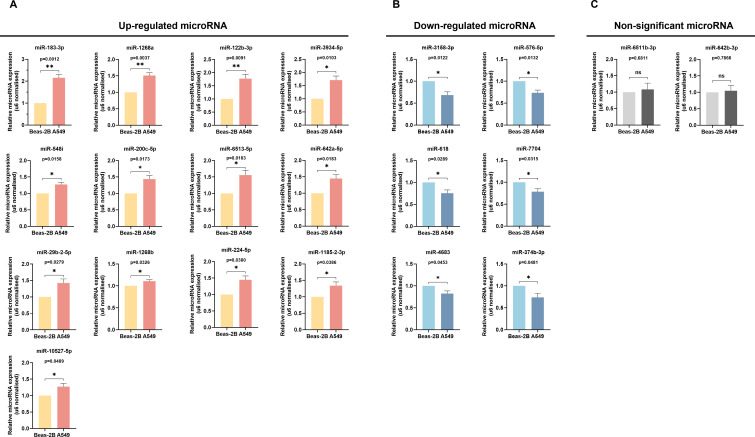
Verification of miRNAs Expression by qRT-PCR. The up-regulated **(A)**, down-regulated **(B)**, and non-significant **(C)** expression of differentially expressed miRNAs between Beas-2B and A549 cells were validated by qRT-PCR. ns, no significance, *p < 0.05, **p < 0.01.

Through an exhaustive literature review, we identified that 15 out of the 36 differentially expressed miRNAs were previously associated with LUAD. To investigate the 21 miRNAs not yet clearly associated with the diagnostics of LUAD further, we conducted qRT-PCR validation to compare their expression in Beas-2B and A549 cells. The qRT-PCR results aligned with the sequencing data for 19 of these 21 miRNAs: 13 were up-regulated (miR-183-3p, miR-1268a, miR-122b-3p, miR-3934-5p, miR-584i, miR-200c-5p, miR-6513-5p, miR-642a-5p, miR-29b-2-5p, miR-1268b, miR-224-5p, miR-1185-2-3p, miR-10527-5p) (**Figure 3A**), 6 were down-regulated (miR-3158-3p, miR-576-5p, miR-618, miR-7704, miR-4683, miR-374b-3p) (**Figure 3B**). Additionally, while the RT-qPCR results for miR-642b-3p followed the same trend as the sequencing data (upregulation in LUAD tissues and A549 cells), there was no significant statistical difference (*p* > 0.05). The RT-qPCR results confirmed miR-6511b-3p levels to be marginally elevated in A549 cells, albeit contrary to the sequencing findings, and had no statistical significance (*p* > 0.05) (**Figure 3C**). These findings indicated our previous sequencing results have robust reliability. Moreover, we pinpointed miR-183-3p as showing the most pronounced differential expression between Beas-2B and A549 cells (*p* = 0.0012) (**Figure 3A**).

### 3.5 Expression level and diagnostic value of differentially expressed miRNAs in LUAD

**Figure 4:**
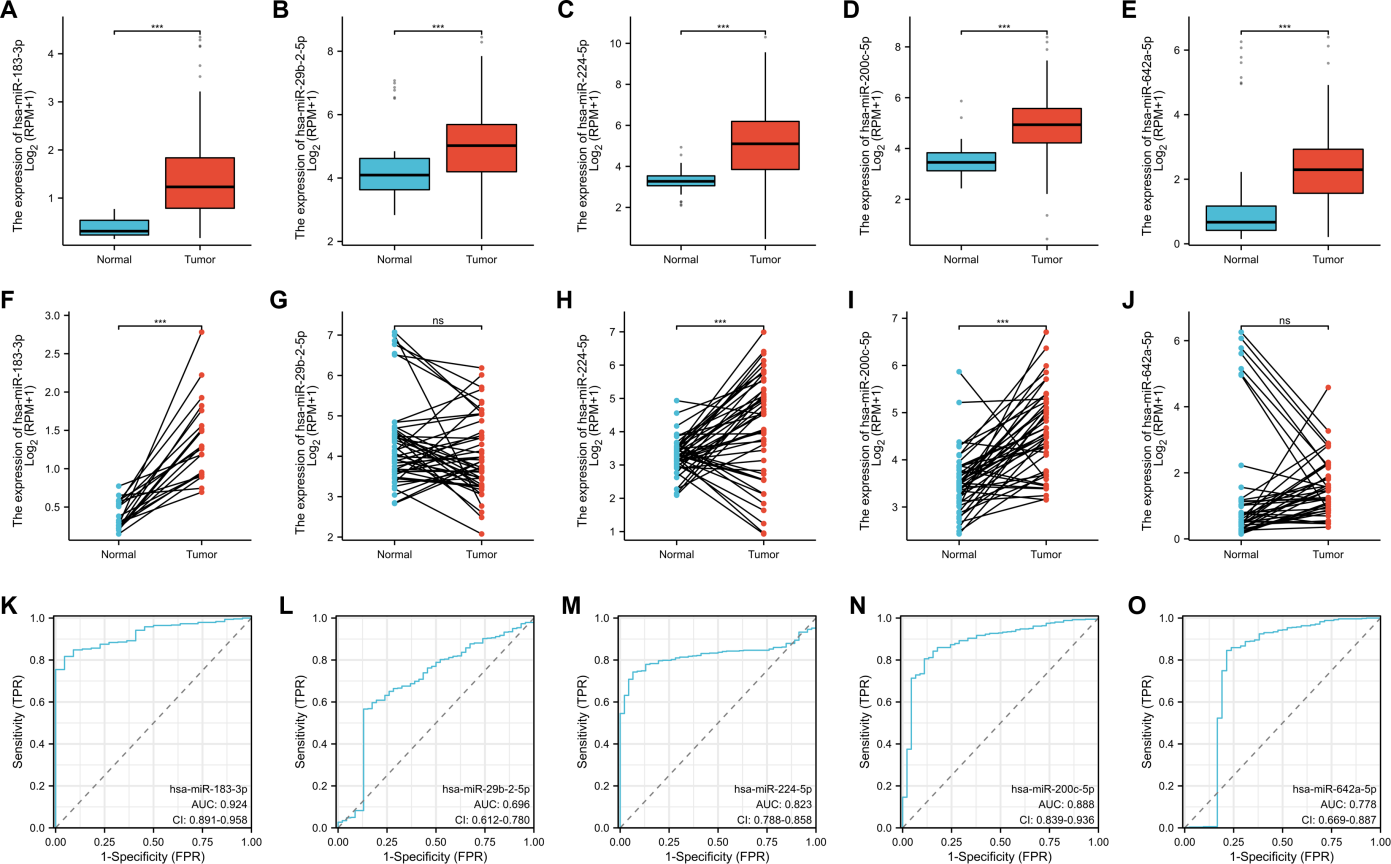
Clinical significance of 5 differentially expressed miRNA in LUAD. The unpaired **(A-E)** and paired **(F-J)** expression of 5 differentially expressed miRNAs between normal and lung cancer tissues. ns, no significance, ***p < 0.01. **(K-O)** ROC analysis of 5 differentially expressed miRNA in LUAD.

In the systematic exploration of the association between all 19 verified differentially expressed miRNAs and the occurrence and prognosis of LUAD, we found five miRNAs were valuable in the clinic: miR-183-3p, miR-29b-2-5p, miR-224-5p, miR-200c-5p, and miR-642a-5p. We evaluated their expression in paired and unpaired tissues. Our findings revealed significant differences in the unpaired tissue expression of all five miRNAs (*p* < 0.001) (**Figures 4A–E**). Notably, miR-183-3p, miR-224-5p, and miR-200c-5p were up-regulated in LUAD tissues compared to their paired normal tissues (*p* < 0.001) (**Figures 4F, H, I**), while miR-642a-5p and miR-29b-2-5p remained relatively no significant difference (**Figures 4G, J**).

Remarkably, all five miRNAs displayed commendable diagnostic predictability based on ROC curve analysis (**Figure 4K–O**). Of these, miR-183-3p displayed the most potent predicting efficacy, with an impressive AUC of 0.924. Given its consistency across sequencing, qRT-PCR data, and clinical relevance, miR-183-3p was chosen for subsequent bioinformatics analysis and functional studies in LUAD cells.

**Figure 5:**
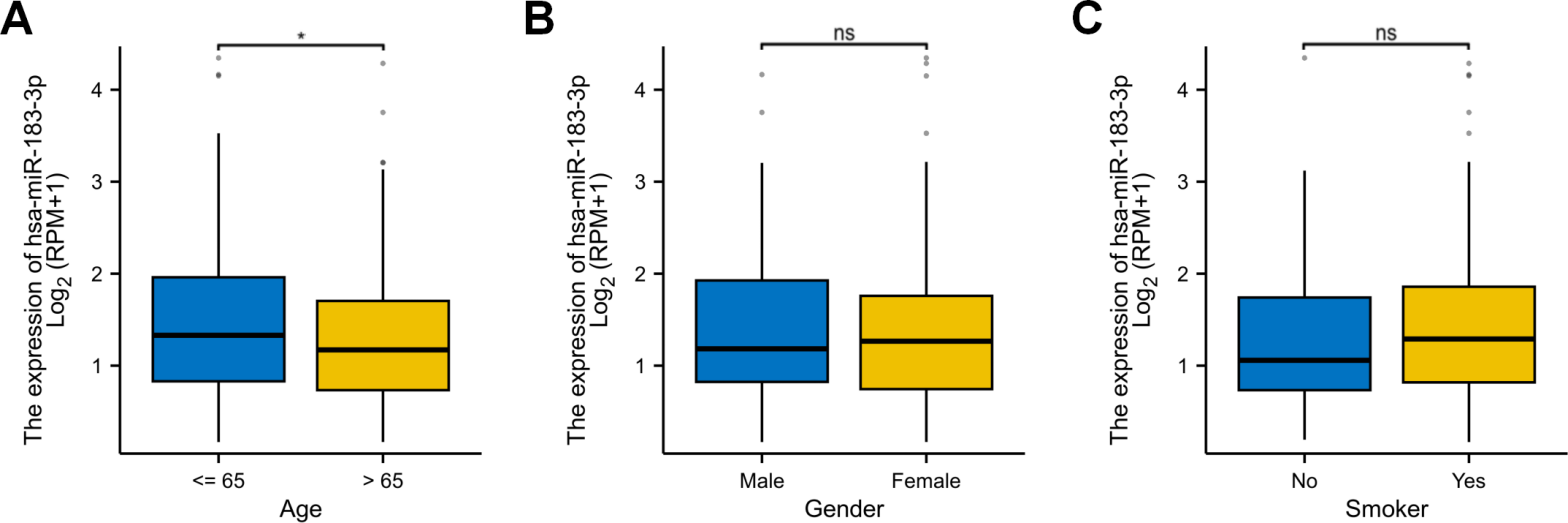
The correlation of miR-183-3p expression with several clinicopathological factors in LUAD. **(A)** Analysis of the correlation between miR-183-3p and the gender of LUAD patients. **(B)** Analysis of the correlation between miR-183-3p and the age of LUAD patients. **(C)** Analysis of the correlation between miR-183-3p and the smoking behavior of LUAD patients. ns, no significance, *p < 0.05.

Analyzing miRNA expression in conjunction with clinicopathological factors such as age, gender, and smoking behavior contributes to a more in-depth exploration of miRNA. We further explored the correlation between miR-183-3p with age, gender, and smoking, utilizing data from the TCGA database. The results similarly demonstrated a significant association with age (*p* < 0.05) (**Figure 5A**) but no significant correlation between miR-183-3p and gender or smoking behavior (*p* > 0.05) (**Figures 5B, C**). These findings shed light on the need for further in-depth research into why age differences result in differences in miR-183-3p expression in LUAD patients.

### 3.6 miR-183-3p inhibition promotes cell apoptosis without affecting cell cycle

**Figure 6:**
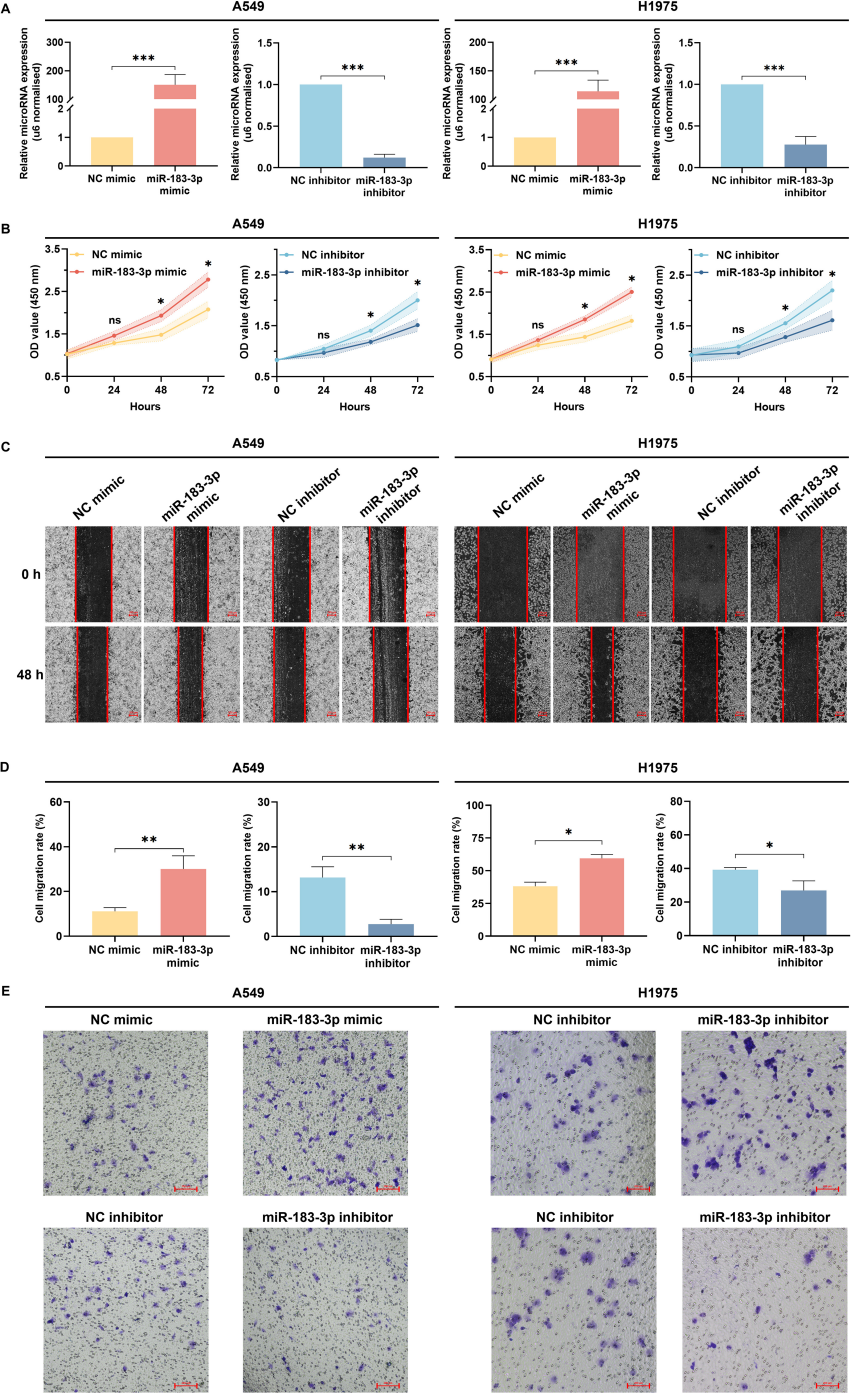
Biological functions of miR-183-3p on the proliferation, migration, and invasion of LUAD cells. **(A)** qRT-PCR was used to detect the transient transfection efficacies of LUAD cells with miR-183-3p mimic and inhibitor. ***p < 0.01. **(B)** CCK-8 assay was used to reflect the proliferation at 0, 24, 48, and 72 h after transfecting with miR-183-3p mimic and inhibitor plasmid in A549 and H1975 cells. The vertical axis represents the OD value of cell proliferation. ns, no significance, *p < 0.05, **p < 0.01. **(C)** A549 and H1975 cells were treated with miR-183-3p mimic and inhibitor vehicle. Wound healing assays were performed, and micrographs were taken 48 h after the scratch. Scale bar = 200 μm. **(D)** Quantification of scratch widths in 4 separate experiments. *p <0.05, **p <0.01 vs. the corre-sponding control group. **(E)** Transwell assays were performed to evaluate the cell invasion. Purple patches indicate invaded cells, whereas small dots indicate the pores on the Matrigel surface.

To investigate the functional impact of miR-183-3p in LUAD cells, synthetic mimics and inhibitors were used to modulate its expression in A549 and H1975 cell lines. Transfection efficacy was confirmed by qRT-PCR, which showed significant up-regulation of miR-183-3p levels with mimic transfection and downregulation with the inhibitor in both cell lines (*p* < 0.01) (**Figure 6A**). Further investigation into miR-183-3p modulation’s cellular effects revealed distinct impacts on cell proliferation. In both A549 and H1975 cells, the overexpression of miR-183-3p significantly promoted cell growth over 48 hours (*p* < 0.05), with a more pronounced effect in A549 cells at 72 hours (*p* < 0.01). On the other hand, the miR-183-3p inhibitor significantly suppresses the proliferation of A549 and H1975 cells at 48 and 72 hours (*p* < 0.05) post-transfection (**Figure 6B**). These findings clarify the role of miR-183-3p in regulating cell proliferation in LUAD cells.

Wound healing assays indicated that cells transfected with miR-183-3p mimic demonstrated expedited closure of wound gaps, indicative of augmented cell migration. In contrast, inhibition of miR-183-3p led to a decrease in cell migration (**Figure 6C**). Quantitative analysis substantiated notable disparities in migration rates among A549 cells (*p* < 0.01) and H1975 cells (*p* < 0.05) in the wound healing assay (**Figure 6D**). Additionally, transwell invasion assays revealed a parallel trend, wherein overexpression of miR-183-3p enhanced the invasive potential of A549 and H1975 cells, whereas its inhibition diminished their invasion capabilities (**Figure 6E**). These findings elucidate that miR-183-3p markedly enhances proliferation, migration, and invasion in LUAD cells, underscoring its critical role in the progression of LUAD.

### 3.7 miR-183-3p enhances proliferation, migration, and invasion in LUAD cells

**Figure 7:**
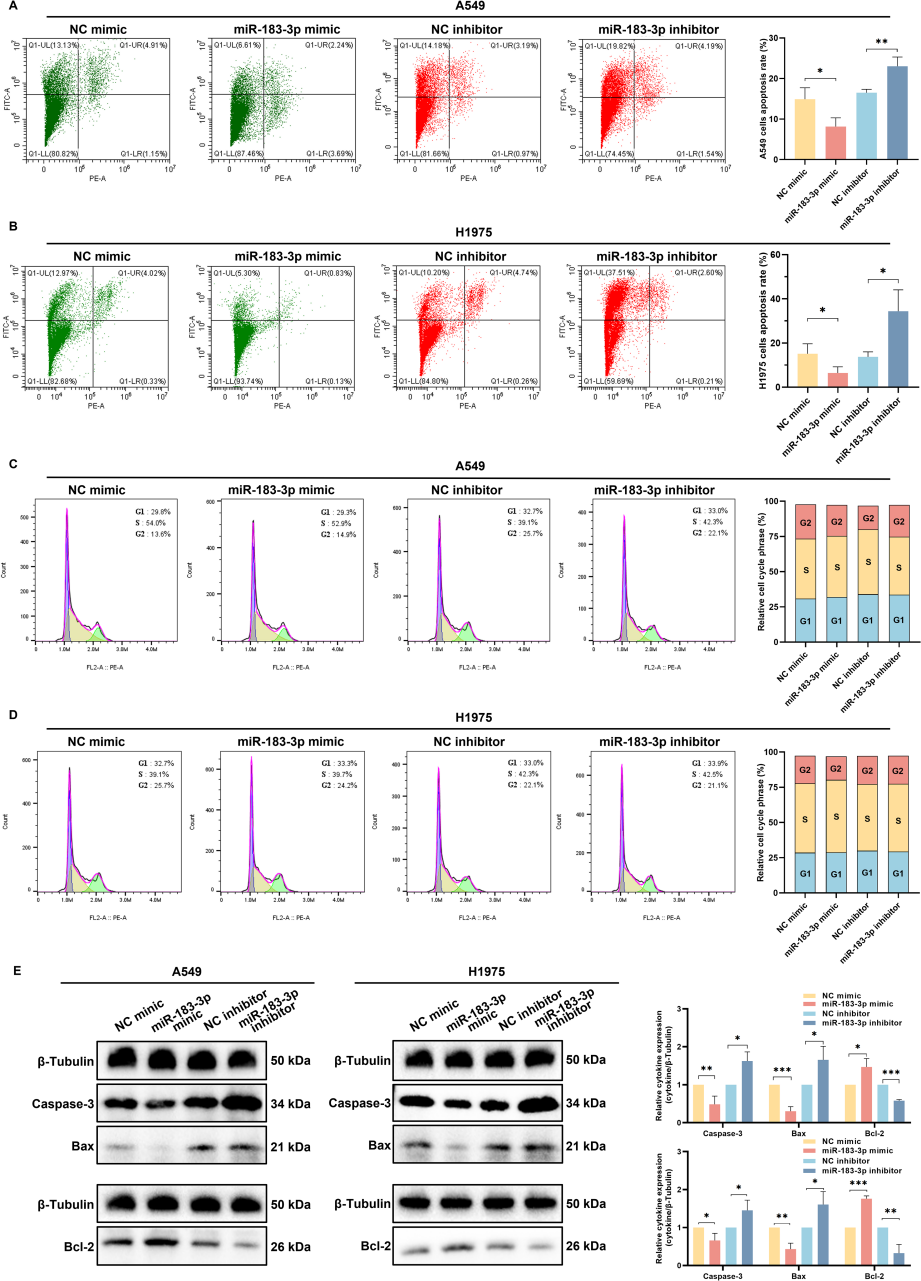
miR-183-3p influences cell apoptosis but not the cell cycle of LUAD cells. **(A, B)** Apoptosis assays for A549 and H1975 cells after transfected with miR-183-3p mimic and inhibitor for 48 h (left panel). Quantitative analysis of miR-183-3p-induced apoptosis, with apoptotic ratios presented as mean ± SD (right panel), *p < 0.05, **p < 0.01. **(C, D)** Cell cycle was detected by flow cytometry (left panel), and the percentage of cells in G1, S, and G2 phases for A549 and H1975 cells (right panel). **(E)** Western blot analysis of A549 and H1975 cells transfected with miR-183-3p mimic and inhibitor for 48 h. Densitometric plot bar graph of Caspase-3, Bax, and Bcl-2 protein expression in overexpression and suppression groups (right panel). The significance level was represented by *p < 0.05, **p < 0.01, ***p < 0.001.

Flow cytometry and quantitative analyses revealed a significant role of miR-183-3p in modulating apoptosis in LUAD cell lines A549 and H1975. Inhibition of miR-183-3p enhanced cell apoptosis, while overexpression impeded cell apoptosis in both cell lines (*p* < 0.05) (**Figures 7A, B**). Additionally, we investigated whether miR-183-3p could regulate the cell cycle in LUAD cells. Our results indicated that modulation of miR-183-3p, either through promotion or suppression, did not significantly alter the cell cycle in A549 and H1975 cells (**Figures 7C, D**).

To further elucidate miR-183-3p’s molecular influence on apoptosis, we assessed the expression of key apoptotic regulators following transfection. Western blot analysis showed transfection with the miR-183-3p inhibitor resulted in increased pro-apoptotic proteins Caspase-3 and Bax levels, and reduced Bcl-2 expression, supporting the pro-apoptotic effect of inhibiting miR-183-3p. Conversely, miR-183-3p mimic transfection led to decreased levels of Caspase-3 and Bax, accompanied by an increase in Bcl-2 expression (*p* < 0.05) (**Figures 7E**). The results reveal a pivotal role for miR-183-3p inhibition in promoting apoptosis in LUAD cells, providing a foundation for developing miR-183-3p-targeted therapeutic strategies in LUAD.

### 3.8 SESN1 as a direct target with clinical relevance of miR-183-3p in LUAD cells

**Figure 8:**
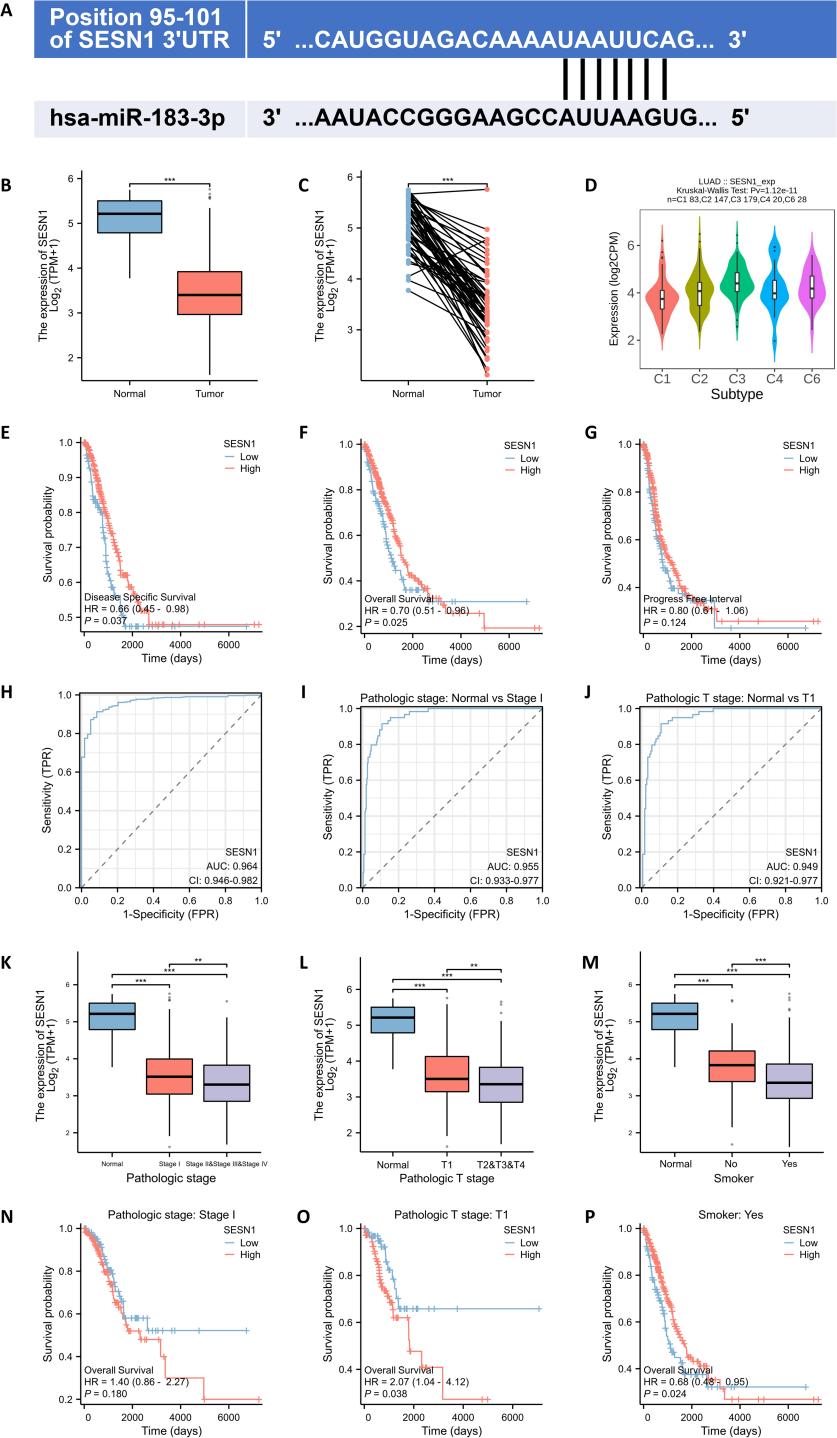
SESN1 is a potential target of miR-183-3p with clinical significance. **(A)** The TargetScan analysis analysis revealed that miR-183-3p binds to the 3’UTR of SESN1. **(B, C)** Comparison of miR-183-3p expression in both paired and unpaired LUAD tissue compared to normal tissue. (Blue: Normal tissues, Red: Tumor samples). ***p < 0.01. **(D)** The association between SESN1 expression and diverse immune subtypes in LUAD (C1: wound healing, C2: IFN-γ dominant, C3: inflammatory, C4: lymphocyte depleted, C5: immunologically quiet, C6: TGF-β dominant). **(E-G)** The correlations between miR-183-3p expression and clinical features in LUAD: **(E)** overall survival; **(F)** disease-specific survival; **(G)** progress-free survival. **(H-J)** ROC curve of SESN1 for LUAD diagnosis, pathological stage and pathological T stage. **(K-P)** Correlation of SESN1 expression with pathologic stage, pathologic T stage, and smoking status, as well as its impact on survival in LUAD. **p < 0.01, ***p < 0.01.

To elucidate the potential molecular mechanisms through which miR-183-3p influences proliferation, apoptosis, invasion, and migration, we utilized TargetScan (http://www.targetscan.org/) databases for predicting the target genes of miR-183-3p. SESN1 was identified as a suitable candidate, attributed to the high complementarity of its mRNA sequence with miR-183-3p (**Figure 8A**). Furthermore, clinical significance analysis of miR-183-3p’s potential target genes showed that the expression level of SESN1 was significantly lower in both paired and unpaired LUAD tissue compared to normal tissue (*p* < 0.001) (**Figures 8B, C**). Additionally, a significant correlation between SESN1 expression and various immune subtypes in LUAD was observed through the Kruskal-Wallis test (*p* = 1.12e-11) (**Figure 7D**). We used the Kaplan-Meier plotter database for survival analysis. Higher expression of SESN1 was associated with better prognosis in overall survival (OS), disease-specific survival (DSS), and progress-free survival (PFS) (**Figures 8E–G**). A ROC analysis was conducted to determine the diagnostic value of SESN1 in LUAD, and the results showed that SESN1 has excellent predictive capabilities (AUC = 0.964) (**Figure 8H**). Additionally, SESN1 exhibited great predictive accuracy in the stage I and T1 stage compared to the normal control (AUC > 0.9) (**Figures 8I, J**). Besides, our study explored the expression level and OS of SESN1 in different stages (**Figures 8K, L, N, O**) and its association with smoking status (**Figures 8M, P**).

**Figure 9:**
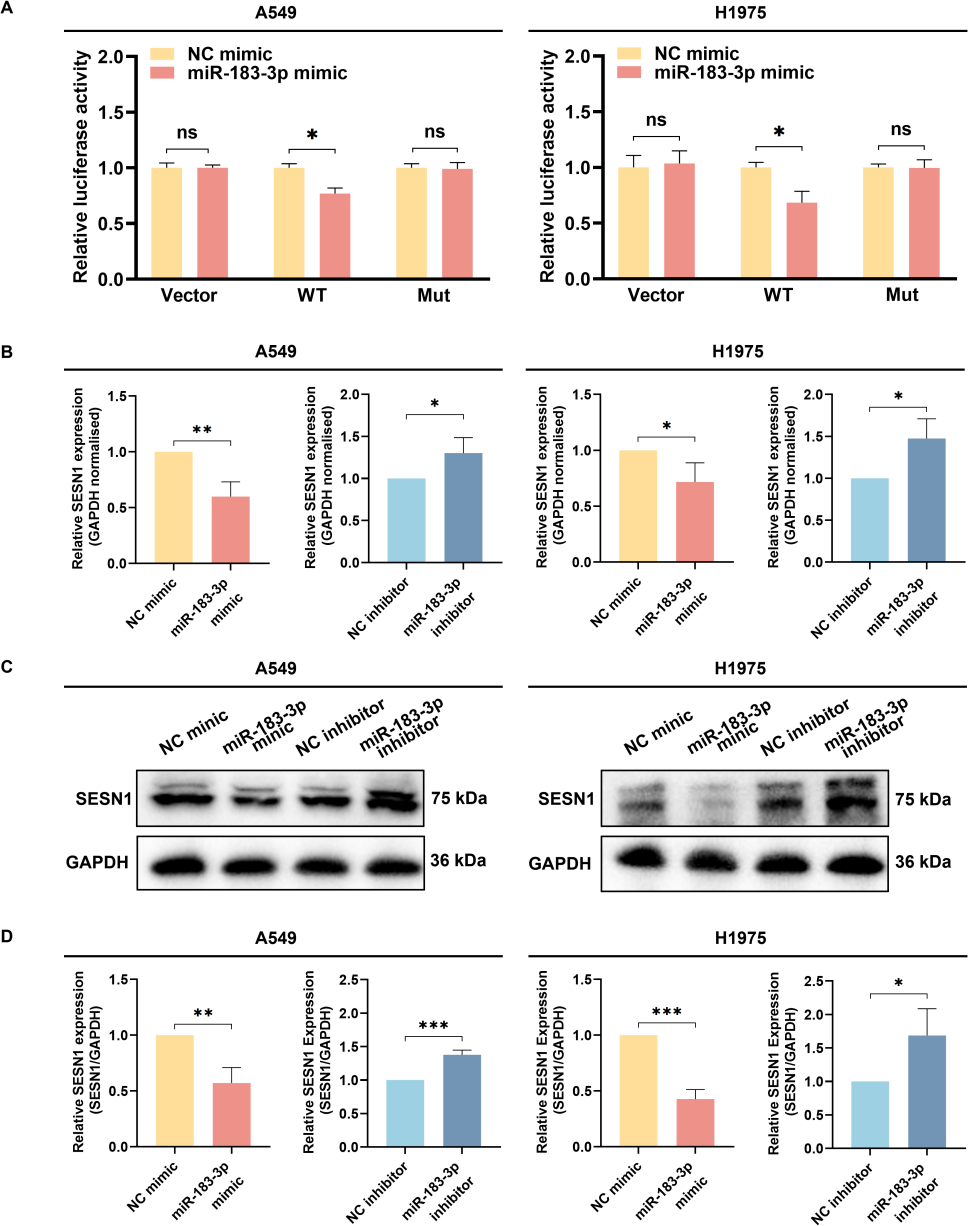
The identification of SESN1 as a target gene of miR-183-3p. **(A)** The luciferase activity was measured following the co-transfection of miR-183-3p mimic with the Vector, SESN1 WT, and SESN1 Mut plasmids. ns, no significance, *p < 0.05. **(B)** The SESN1 mRNA expression in A549 and H1975 cells with miR-183-3p mimic and inhibitor plasmid. *p < 0.05, **p < 0.01. **(C)** The SESN1 protein expression in LUAD cells with miR-183-3p mimic and inhibitor was measured by Western blot. **(D)** Histograms depict the intensity of SESN1 protein expression in both A549 and H1975 cells. ns, no significance, *p < 0.05, **p < 0.01, ***p < 0.01.

Target gene validation was performed using luciferase reporter assays, qRT-PCR, and western blotting. Luciferase reporter assays demonstrated that miR-183-3p over-expression significantly reduced the luciferase activity of a reporter containing the wild-type 3’-UTR of SESN1 (*p* < 0.05). This suppressive effect was absent when the miR-183-3p binding site was mutated, underscoring the specificity of this interaction (**Figure 9A**). Our findings indicated that overexpression of miR-183-3p substantially diminishes both mRNA (*p* < 0.05) and protein (*p* < 0.05) levels of SESN1 in A549 and H1975 cells. In contrast, inhibition of miR-183-3p leads to a significant upregulation of SESN1 expression at both the mRNA (*p* < 0.05) and protein (*p* < 0.05) levels (**Figures 9B–D**). Furthermore, These observations highlight SESN1 as a direct and functional target of miR-183-3p in LUAD cells, suggesting that miR-183-3p may promote LUAD progression via SESN1 suppression. This molecular interaction delineates a potential pathway by which miR-183-3p contributes to the oncogenic phenotype in LUAD.

## 4 Discussion

**Figure 10:**
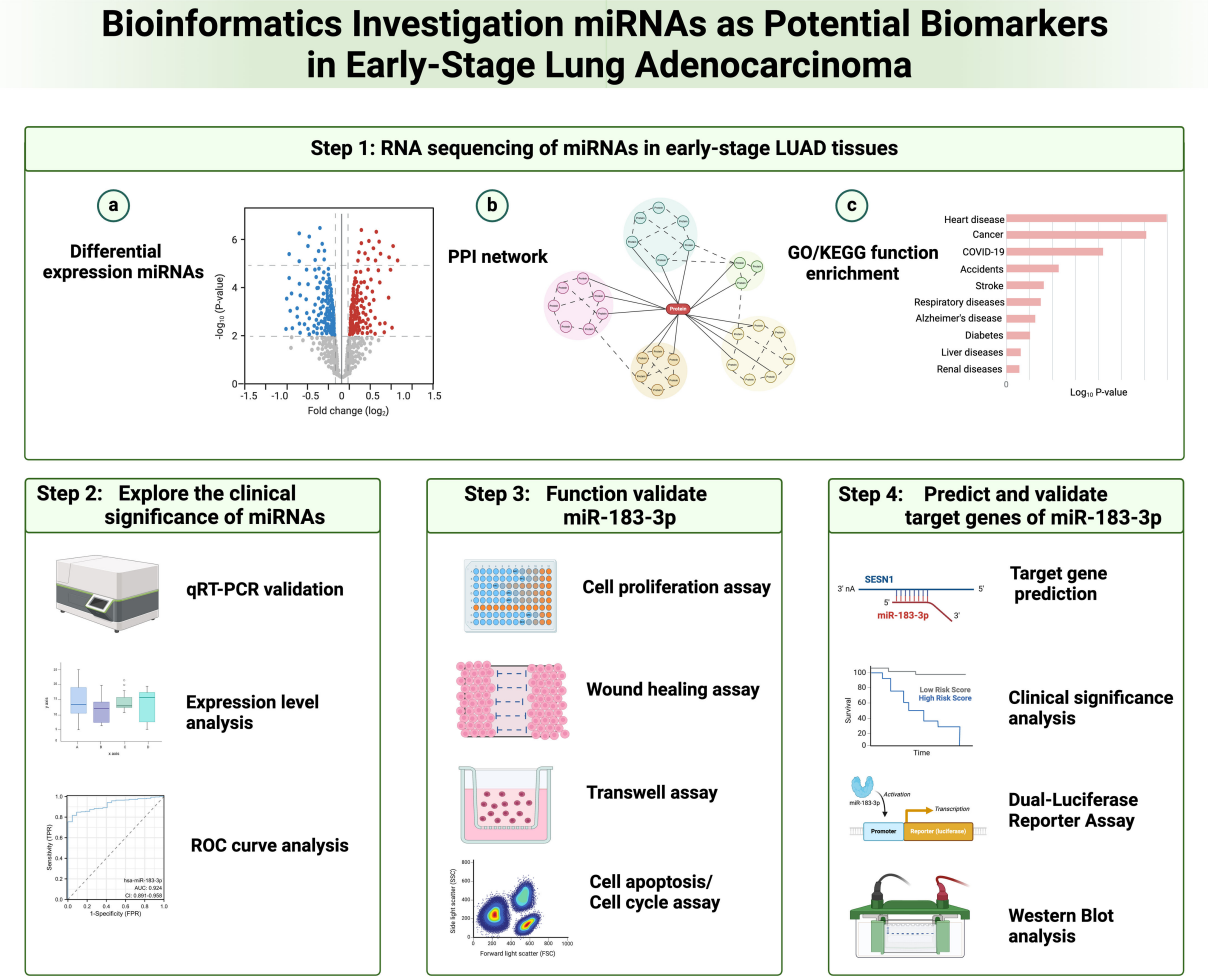
Flow diagram for integration analysis of microRNAs as potential biomarkers in early-stage lung adenocarcinoma.

Lung cancer’s development is intricate, making it challenging for any single gene, protein, or pathway to encapsulate its pathogenesis fully. Recently, several therapeutic approaches have emerged for lung cancer treatment, notably molecular targeting drugs, anti-vascular drugs, and immune-targeted drugs. Among these, the utilization of molecular targeting drugs has notably boosted the 5-year survival rate for LUAD patients (27). Past research has pinpointed the aberrant expression of miRNAs as a key factor in lung cancer’s onset and progression (28). Given this, miRNAs are increasingly being recognized as potential biomarkers for lung cancer, and our integration analysis provided insight into miRNAs’ diagnostic and therapeutic significance of lung adenocarcinoma (**Figure 10**).

Our research harnessed the power of RNA-seq, a cutting-edge approach grounded in next-generation sequencing technology. At its core, RNA-seq conducts expansive, parallel sequencing of the transcriptome RNA, enabling both qualitative and quantitative assessment of all RNAs through rigorous data analysis (29). This technique markedly outperforms conventional microarray technology in terms of both quality and quantity.

The miR-183 cluster presents abnormal expression across various cancers, hinting at its broad influence on tumor growth. However, its expression has been shown to be inconsistent (30). Specifically, miR-183-5p has been pinpointed as a key modulator of lung cancer cell proliferation and migration. Wang H et al. provided evidence that miR-183-5p plays a pivotal role in NSCLC progression by targeting PTEN. Subsequent experiments revealed miR-183-5p’s suppression of p53 and activation of AKT signaling via phosphorylation (9). Additionally, miR-183-5p was found to boost the epithelial-mesenchymal transition (EMT) in LUAD cells, leading to enhanced radiation resistance (31). Yet, in a twist, Meng F et al. identified miR-183-5p as a potential tumor suppressor in lung cancer, operating through PIK3CA (7). Such contradictory findings are mirrored in studies on colorectal, prostate, and gastric cancers (10, 32–36). Xu F et al. observed miR-183-3p overexpression specifically in LUAD from female non-smokers (11). Our findings spotlight an upregulation of miR-183-3p in initial-stage LUAD issues and its role in bolstering biological functions. Nevertheless, the underlying processes remain enigmatic. Drawing from our past research, we anticipate subsequent studies will shed clearer light on the nuanced regulatory impacts of the miR-183 cluster in tumor genesis and evolution.

SESN1 belongs to the SESN family, known for its tumor-suppressing abilities. Both SESN1 and SESN2 are transcriptionally regulated by p53 and amplify the tumor suppressor effects of p53. Upon cellular stress or DNA damage, activated p53 binds to specific sequences in the promoters of SESN1 and SESN2 genes, culminating in their transcriptional enhancement. Notably, SESN1 and SESN2 serve as pivotal inhibitors within the mTOR pathway. When p53 is activated by genotoxic and oxidative stress, it inhibits cell proliferation and growth through the induction of specific target genes, including SESN 1 and SESN 2. These Sestrin gene products were found to activate AMPK (AMP-responsive protein kinase) and direct it to phosphorylate TSC2, thereby stimulating TSC2’s GAP activity and inhibiting mTOR signaling. The research also showed that SESN2-deficient mice were unable to inhibit mTOR signaling in response to genotoxic challenges. Therefore, SESN1 are identified as important mediators linking genotoxic stress, p53, and the mTOR signaling pathway, which plays a central role in regulating cell growth and survival (37). What’s more, SESN1 can induce anti-stress responses, including the inhibition of cell proliferation and adjustment to fluctuating nutrient conditions by inhibiting mTORC1, enhancing autophagy/mitophagy to maintain organelle quality, and instigating an unfolded protein response to impede the aggregation of misfolded proteins (38). A reduced expression of SESN2 correlates with a less favorable prognosis in colorectal cancer (39). Studies have portrayed SESN2 as a central regulator of mTORC1 signaling, inhibiting colon cancer development (40). Moreover, SESN2-lacking mouse embryonic fibroblasts exhibited a higher vulnerability to oncogenic transformation compared to their wild-type peers, implying a tumor-suppressing role for SESN2 (41). Disabling SESN1 and/or SESN2 in A549 cells amplifies cell growth and bestows resistance to cell death under glucose scarcity, thereby fostering early tumor expansion (42). Our research indicates that miR-183-3p directly curtails SESN1 expression, suggesting a significant role for SESN1 in LUAD progression.

However, several limitations of our study should be noted. We acknowledge the limitation regarding the small sample size of patients, which was due to several factors. Firstly, in order to identify microRNAs with significantly differential expression in the early stages of LUAD, we implemented strict inclusion criteria, focusing only on patients with early-stage LUAD. Additionally, our study was constrained by a limited duration, which restricted the number of patients we could recruit. Furthermore, our sample collection was conducted at a single center, which further limited the sample size. We recommend that other researchers extend their study duration and conduct research across multiple centers in future explorations of biomarkers. This approach will enhance the reliability of research conclusions.

The intricacies of cellular heterogeneity and the molecular machinations propelling LUAD progression have long remained obscure. Our study offers an in-depth analysis of transcriptomes from LUAD and adjacent non-tumor tissues in 33 patients, providing a panoramic view of the molecular landscape. Harnessing RNA-seq technology, we identified numerous miRNAs with differential expression in the nascent stages of LUAD. The discoveries from our work illuminate the differential expression of miRNA in early histological phases of LUAD progression and furnish potential molecular indicators for the clinical detection and management of incipient LUAD. Our research highlights miR-183-3p, along with four other miRNAs, as potential clinical diagnostic markers for early-stage LUAD. Furthermore, miR-183-3p directly re-presses SESN1 and plays the biological function of promoting cancer in LUAD cells. Inhibiting miR-183-3p offers potential as a promising therapeutic strategy for LUAD.

## Data Availability

The raw data supporting the conclusions of this article will be made available by the authors, without undue reservation.
