# Supplementary material for: Integration analysis of microRNAs as potential biomarkers in early-stage lung adenocarcinoma: the diagnostic and therapeutic significance of miR-183-3p
Source: Front Oncol. 2024 Dec 17;14:1508715. doi: 10.3389/fonc.2024.1508715 (PMC11697600; doi:10.3389/fonc.2024.1508715)
Supplement: Supplementary file 1 [file Table1.docx]

Supplementary Material

**Integration analysis of microRNAs as potential biomarkers in early-stage lung adenocarcinoma: the diagnostic and biological significance of miR-183-3p**

**Guodong Huang^1†^, Yuxia Liu^2†^, Lisha Li^3†^, Bing Li^1^, Ting Jiang^1^, Yufeng Cao^4^, Xiaoping Yang^3^, Xinning Liu^1^, Honglin Qu^1^, Shitao Li^2*^ and Xin Zheng^1*^**

**Correspondence:** Xin Zheng: [zyxy66999@163.com](mailto:zyxy66999@163.com); Shitao Li [shitaosd@hotmail.com](mailto:shitaosd@hotmail.com)

# Supplementary Tables

## Supplementary Table 1. The specific primer sequences of miRNA and mRNA in qRT-PCR

| Number | miRNA or mRNA | Sequencing |
| --- | --- | --- |
| 1 | hsa-miR-183-3p (F) | GTGAATTACCGAAGGGCCATAA |
| 2 | hsa-miR-1268a (F) | GGCGGGCGTGGTGGTGGGGG |
| 3 | hsa-miR-122b-3p (F) | AAACACCATTGTCACACTCCAC |
| 4 | hsa-miR-3934-5p (F) | GCCAGCTCCTACATCTCAGC |
| 5 | hsa-miR-548i (F) | GGGAAAAGTAATTGCGGATTTTGCC |
| 6 | hsa-miR-200c-5p (F) | CGTCTTACCCAGCAGTGTTTGG |
| 7 | hsa-miR-6513-5p (F) | CGGTTTGGGATTGACGCCACATG |
| 8 | hsa-miR-642a-5p (F) | GTCCCTCTCCAAATGTGTCTTG |
| 9 | hsa-miR-29b-2-5p (F) | CTGGTTTCACATGGTGGCTTAG |
| 10 | hsa-miR-1268b (F) | CGGGCGTGGTGGTGGGGGTG |
| 11 | hsa-miR-224-5p (F) | CAAGTCACTAGTGGTTCCGTTTAG |
| 12 | hsa-miR-1185-2-3p (F) | ATATACAGGGGGAGACTCTCAT |
| 13 | hsa-miR-10527-5p (F) | AAAGCAAATGTTGGGTGAACGGC |
| 14 | hsa-miR-3158-3p (F) | AAGGGCTTCCTCTCTGCAGGAC |
| 15 | hsa-miR-576-5p (F) | ATTCTAATTTCTCCACGTCTTT |
| 16 | hsa-miR-618 (F) | AAACTCTACTTGTCCTTCTGAGT |
| 17 | hsa-miR-7704 (F) | CGGGGTCGGCGGCGACGTG |
| 18 | hsa-miR-4683 (F) | AGATCCAGTGCTCGCCCGAT |
| 19 | hsa-miR-374b-3p (F) | CTTAGCAGGTTGTATTATCATT |
| 20 | hsa-miR-6511b-3p (F) | CACCACCCCTTCTGCCTGCA |
| 21 | hsa-miR-642a-5p (F) | GTCCCTCTCCAAATGTGTCTTG |
| 22 | U6 (F) | GGGCAGGAAGAGGGCCTAT |
| 23 | SESN1 (F) | TGCTTTGGGCCGTTTGGATAA |
| 24 | SESN1 (R) | TGTAGTGACGATAATGTAGGGGT |
