# Supplementary material for: Integration analysis of microRNAs as potential biomarkers in early-stage lung adenocarcinoma: the diagnostic and therapeutic significance of miR-183-3p
Source: Front Oncol. 2024 Dec 17;14:1508715. doi: 10.3389/fonc.2024.1508715 (PMC11697600; doi:10.3389/fonc.2024.1508715)
Supplement: Supplementary file 2 [file Table2.docx]

Supplementary Material

**Integration analysis of microRNAs as potential biomarkers in early-stage lung adenocarcinoma: the diagnostic and biological significance of miR-183-3p**

**Guodong Huang^1†^, Yuxia Liu^2†^, Lisha Li^3†^, Bing Li^1^, Ting Jiang^1^, Yufeng Cao^4^, Xiaoping Yang^3^, Xinning Liu^1^, Honglin Qu^1^, Shitao Li^2*^ and Xin Zheng^1*^**

**Correspondence:** Xin Zheng: [zyxy66999@163.com](mailto:zyxy66999@163.com); Shitao Li [shitaosd@hotmail.com](mailto:shitaosd@hotmail.com)

# Supplementary Tables

## Supplementary Table 2. The up-regulated miRNAs in lung cancer and paracancerous tissues.

| Ranking | ID | Sequence | Log_2_FC | *P* value |
| --- | --- | --- | --- | --- |
| 1 | hsa-miR-183-3p | AAUACCGGGAAGCCAUUAAGUG | 4.989894761 | 9.81E-41 |
| 2 | hsa-miR-3617-5p | GGGUAGAACGUUGAUACAGAAA | 3.257698298 | 1.41E-21 |
| 3 | hsa-miR-642b-3p | CCCAGGGAGAGGUUUACACAGA | 3.248337193 | 1.96E-20 |
| 4 | hsa-miR-708-3p | GAUCUUCGAGUGUCAGAUCAAC | 3.002789242 | 4.70E-16 |
| 5 | hsa-miR-323b-3p | UUCUCCAGCUGGCACAUAACCC | 2.975158359 | 5.34E-19 |
| 6 | hsa-miR-548i | CCGUUUUAGGCGUUAAUGAAAA | 2.939660973 | 2.07E-09 |
| 7 | hsa-miR-25-5p | GUUAACGGGUUCAGAGGCGGA | 2.626349645 | 1.97E-14 |
| 8 | hsa-miR-200c-5p | GGUUUGUGACGACCCAUUCUGC | 2.193028006 | 6.56E-10 |
| 9 | hsa-miR-1268a | GGGGGUGGUGGUGCGGGC | 2.153431797 | 5.56E-07 |
| 10 | hsa-miR-1268b | GUGGGGGUGGUGGUGCGGGC | 2.136656699 | 8.09E-07 |
| 11 | hsa-miR-10527-5p | CGGCAAGUGGGUUGUAAACGAAA | 1.983309543 | 2.13E-11 |
| 12 | hsa-miR-29b-2-5p | GAUUCGGUGGUACACUUUGGUC | 1.975541391 | 3.30E-07 |
| 13 | hsa-miR-130b-5p | CAUCACGUUGUCCCUUUCUCA | 1.957891137 | 1.09E-20 |
| 14 | hsa-miR-4677-3p | UCAUCAAGAAACCAGAGUGUCU | 1.644500449 | 8.29E-07 |
| 15 | hsa-miR-122b-3p | CACCUCACACUGUUACCACAAA | 1.625005784 | 1.61E-13 |
| 16 | hsa-miR-629-3p | CGACCCGAAUGCAACCCUCUUG | 1.616864631 | 4.05E-09 |
| 17 | hsa-miR-934 | GGUCACAGAGGUCAUCAUCUGU | 1.560515792 | 0.012221868 |
| 18 | hsa-miR-3934-5p | GACGGAGUCAAAGGUGUGGACU | 1.539690621 | 6.61E-07 |
| 19 | hsa-miR-642a-5p | GUUCUGUGUAAACCUCUCCCUG | 1.447498758 | 1.50E-10 |
| 20 | hsa-miR-1185-2-3p | UACUCUCAGAGGGGGACAUAUA | 1.44676729 | 0.002461558 |
| 21 | hsa-miR-625-5p | CCUGAUAUCUUGAAAGGGGGA | 1.422620519 | 5.79E-11 |
| 22 | hsa-miR-296-3p | CCUCUCGGAGGUGGGUUGGGAG | 1.2437673 | 1.67E-05 |
| 23 | hsa-miR-21-3p | UGUCGGGUAGCUGACCACAAC | 1.238133357 | 4.85E-18 |
| 24 | hsa-miR-224-5p | GAUUUGCCUUGGUGAUCACUGAACU | 1.034329038 | 1.06E-09 |
