# Supplementary material for: Integration analysis of microRNAs as potential biomarkers in early-stage lung adenocarcinoma: the diagnostic and therapeutic significance of miR-183-3p
Source: Front Oncol. 2024 Dec 17;14:1508715. doi: 10.3389/fonc.2024.1508715 (PMC11697600; doi:10.3389/fonc.2024.1508715)
Supplement: Supplementary file 3 [file Table3.docx]

Supplementary Material

**Integration analysis of microRNAs as potential biomarkers in early-stage lung adenocarcinoma: the diagnostic and biological significance of miR-183-3p**

**Guodong Huang^1†^, Yuxia Liu^2†^, Lisha Li^3†^, Bing Li^1^, Ting Jiang^1^, Yufeng Cao^4^, Xiaoping Yang^3^, Xinning Liu^1^, Honglin Qu^1^, Shitao Li^2*^ and Xin Zheng^1*^**

**Correspondence:** Xin Zheng: [zyxy66999@163.com](mailto:zyxy66999@163.com); Shitao Li [shitaosd@hotmail.com](mailto:shitaosd@hotmail.com)

# Supplementary Tables

## Supplementary Table 3. The down-regulated miRNAs in lung cancer and paracancerous tissues

| Ranking | ID | Sequence | Log_2_FC | *P* value |
| --- | --- | --- | --- | --- |
| 1 | hsa-miR-618 | UGAGUCUUCCUGUUCAUCUCAAA | -3.026193387 | 8.65E-17 |
| 2 | hsa-miR-3158-3p | CAGGACGUCUCUCCUUCGGGAA | -2.852305312 | 1.11E-12 |
| 3 | hsa-miR-4683 | UAGCCCGCUCGUGACCUAGAGGU | -2.131115634 | 1.27E-10 |
| 4 | hsa-miR-7704 | GUGCAGCGGCGGCUGGGGC | -1.976727785 | 4.57E-09 |
| 5 | hsa-miR-584-5p | UUAUGGUUUGCCUGGGACUGAG | -1.481050698 | 0.001317705 |
| 6 | hsa-miR-6513-5p | UCUGUACACCGCAGUUAGGGUUU | -1.367849303 | 4.61E-05 |
| 7 | hsa-miR-3615 | CUCGGCGCUCCUCGGCUCUCU | -1.360427466 | 0.008765963 |
| 8 | hsa-miR-374b-3p | UUACUAUUAUGUUGGACGAUUC | -1.323494515 | 0.022697229 |
| 9 | hsa-miR-6511b-3p | ACGUCCGUCUUCCCCACCACUCC | -1.2137949 | 0.005108266 |
| 10 | hsa-miR-576-5p | UUUCUGCACCUCUUUAAUCUUA | -1.165760133 | 0.001474318 |
| 11 | hsa-miR-1247-3p | CGAGGUCAGAGCUGCAAGGGCCCC | -1.137948264 | 0.000438172 |
| 12 | hsa-miR-2110 | GUGAGUCGCCGGCAAAGGGGUU | -1.091163091 | 0.011139249 |
